# Linking ABO blood group with angle's classification and malocclusion severity in orthodontic patients: A cross-sectional study

**DOI:** 10.6026/973206300212120

**Published:** 2025-07-31

**Authors:** Zeeshan Mubashir, Heena Dixit, Akhilesh M., Arti Devi, Subash Ch. Nayak, Dhananjay Rathod, Deepak Sharma

**Affiliations:** 1 Department of Orthodontic and Dentofacial Orthopaedics, Al Jouf specialised dental center, Ministry of Health, Sakaka, Aljouf Province Kingdom of Saudi Arabia; 2 Department of Medical Health Administration, Index Institute, Malwanchal University, Index City, Nemawar Road, Indore, Madhya Pradesh, India; 3 Department of Orthodontics and Dentofacial Orthopaedics, Consultant Orthodontist, Hyderabad, India; 4 Department of Orthodontics & Dentofacial Orthopaedics, MMCDSR, Mullana, Ambala, Haryana, India; 5 Department of Orthodontics & Dentofacial Orthopedics, Hi-Tech Dental College & Hospital, Bhubaneswar, India; 6 Department of Dentistry, Sheikh Bhikhari Medical College, Hazaribagh, Jharkhand, India

**Keywords:** ABO blood group, malocclusion, angle's classification, orthodontics, IOTN

## Abstract

ABO blood group antigens, known to play roles beyond immunohematology, may contribute to craniofacial development and dental arch alignment. A cross-sectional research was conducted among 200 orthodontic patients aged 12-30 years. Blood group O showed a higher prevalence of Class I malocclusion (48.9%), while Class II was more common in group B (46.3%). Class III showed increased occurrence in group A (41.7%). A statistically significant association was found between ABO blood group and both malocclusion type (p=0.043) and severity (p=0.038). ABO blood group may be a predictive marker in malocclusion type and severity.

## Background:

The role of genetics in craniofacial development and dental occlusion has long intrigued orthodontic researchers. Among the various hereditary traits, the ABO blood group system represents a widely studied marker that may potentially correlate with different phenotypic expressions, including dental and skeletal characteristics. While blood groups have been explored for associations with systemic conditions, their relationship with oral health, especially malocclusion, remains a relatively underexplored area. Recent scientific inquiries suggest that blood group antigens, which are expressed not only on erythrocytes but also on various epithelial cells, may influence the development of oral tissues and structures during embryogenesis [1, 2-3]. Angle's classification remains the most universally accepted system to describe the sagittal relationships of the dental arches and has proven to be a valuable clinical tool in orthodontic diagnosis. Malocclusion, which encompasses deviations from the ideal occlusion, is influenced by a complex interplay of genetic, environmental and epigenetic factors. The etiology of malocclusion has conventionally centered on jaw size discrepancies, muscular dysfunction and oral habits, but the potential contribution of genetic markers such as ABO blood groups presents a novel perspective [4, 5-6]. Cross-sectional epidemiological studies conducted across various populations have yielded conflicting outcomes. Some have proposed that certain blood groups may predispose individuals to specific types of malocclusion, while others have failed to establish any significant association [7, 8-9]. This discrepancy may stem from population-specific genetic variations, environmental modifiers, and sample size limitations. Nevertheless, the hypothesis that the ABO blood group system could be associated with the frequency or severity of malocclusion merits further validation, particularly in diverse demographic contexts. This research aims to investigate the correlation between ABO blood groups and the type and severity of malocclusion based on Angle's classification in orthodontic patients. A better understanding of such a correlation could offer novel insights into individualized risk profiling and early interception strategies in orthodontics [10]. Therefore, it is of interest to study ABO blood group correlation with angle's classification and malocclusion severity in orthodontic patients.

## Materials and Methods:

## Research design and setting:

This was an observational, cross-sectional research conducted in the Department of Orthodontics at a tertiary dental institution for 12 months. Ethics approval and informed consent was obtained from all participants. A total of 200 orthodontic patients aged between 12 and 30 years, who reported for orthodontic consultation and had not undergone previous orthodontic treatment, were included. Participants were designated using consecutive sampling based on the selection methods. Patients with craniofacial anomalies, syndromes, systemic diseases, or those who had received any form of orthodontic therapy were excluded to avoid confounding variables. Demographic data such as age and gender were recorded. Blood group typing (ABO and Rh factor) was obtained from medical records or confirmed through a slide agglutination test using standard monoclonal antisera. Occlusal relationships were assessed clinically by a calibrated orthodontist using Angle's classification (Class I, II, and III), and malocclusion severity was graded using the Index of Orthodontic Treatment Need (IOTN). Using the SPSS ver 26, the data was analysed and p-value of <0.05 was considered statistically significant. Intra-examiner reliability was assessed through repeated examination of 10% of the sample after two weeks, yielding a kappa value of 0.88, indicating high agreement.

## Results:

A total of two hundred orthodontic patients were included in the research, with a mean age of 18.6 ± 4.3 years. The gender distribution showed a slight female predominance (58% female, 42% male). The most common blood group observed was O (38%), followed by B (30%), A (22%) and AB (10%). Based on Angle's classification, Class I malocclusion was most prevalent (47%), followed by Class II (41%) and Class III (12%). Analysis revealed that Class I malocclusion was more commonly seen in individuals with blood group O (52.6%), while Class II was more frequent among those with blood group B (46.7%). Class III malocclusion had a relatively higher proportion among individuals with blood group A (20.5%). The association between blood group and Angle's classification was found to be statistically significant (p = 0.043), suggesting a potential correlation (Table 1 - see PDF). When malocclusion severity was assessed using the IOTN, 36.5% of blood group B patients exhibited severe malocclusion compared to 18.6% of those with blood group O. Moderate severity was seen across all blood groups, with mild cases being least common. A statistically significant correlation was noted between blood group and malocclusion severity (p = 0.038). These findings suggest a statistically significant association between ABO blood groups and both the type and severity of malocclusion among orthodontic patients (Table 2 - see PDF).

## Discussion:

The present research aimed to explore the correlation between ABO blood groups and malocclusion types as classified by Angle, as well as the severity of malocclusion determined by IOTN grading. The results revealed a statistically significant association between specific blood groups and both the type and severity of malocclusion, suggesting a potential genetic or developmental link. The predominance of Class I malocclusion observed in individuals with blood group O supports earlier hypotheses suggesting that individuals with this blood group may have relatively balanced craniofacial development [11]. Conversely, Class II malocclusion showed a higher frequency among those with blood group B, while Class III malocclusion was more common in individuals with blood group A. These findings align with certain prior reports indicating blood group-based variations in dental arch relationships, although other studies have reported contradictory results [12]. The underlying mechanisms behind such associations may lie in embryological development. ABO blood group antigens are expressed not only on red blood cells but also on epithelial and endothelial cells during morphogenesis. These antigens could influence maxillofacial tissue differentiation and growth patterns, potentially predisposing individuals to specific occlusal traits [13]. Additionally, blood group antigens may modulate immune responses or interact with salivary proteins that contribute indirectly to oral and dental development. Regarding malocclusion severity, this research found that individuals with blood group B had a higher proportion of severe malocclusion cases, whereas mild cases were more frequent among those with blood group O. These findings reinforce the concept that certain blood groups may be predictive of more complex orthodontic treatment needs. This could be particularly useful for early risk identification and developing individualized preventive or interceptive strategies. Despite the significant associations identified, this research is not without limitations. The sample size was modest and derived from a single geographic location, which may limit generalizability. Furthermore, environmental factors such as socio-economic status, and dietary habits, oral hygiene practices, were not controlled, and these could act as confounders. Future research with larger, multi centric populations and genomic profiling may yield deeper insights into these correlations [14, 15]. Overall, the findings provide a foundation for exploring the ABO blood group system as a potential risk marker in orthodontic diagnosis and planning. While not definitive, this genetic trait may serve as one among several factors to consider in a comprehensive orthodontic assessment [16, 17, 18, 19-20].

## Conclusion:

Blood group O was more commonly associated with Class I and milder forms of malocclusion, while blood group B was linked with higher occurrences of Class II and severe malocclusion cases. Incorporating blood group profiling in early orthodontic evaluations may aid in risk stratification and individualized treatment approaches, although further large-scale studies are required for validation.
